# Hide and Seek: The Interplay Between Zika Virus and the Host Immune Response 

**DOI:** 10.3389/fimmu.2021.750365

**Published:** 2021-10-21

**Authors:** Lim Jack Lee, Thamil Vaani Komarasamy, Nur Amelia Azreen Adnan, William James, Vinod RMT Balasubramaniam

**Affiliations:** ^1^ Infection and Immunity Research Strength, Jeffrey Cheah School of Medicine and Health Sciences, Monash University Malaysia, Bandar Sunway, Malaysia; ^2^ Sir William Dunn School of Pathology, University of Oxford, Oxford, United Kingdom

**Keywords:** Zika virus, congenital abnormalities, immunopathogenesis, innate and adaptive immunity, sexual transmission, pregnancy, cross-reactive immunity, vaccine

## Abstract

Zika virus (ZIKV) received worldwide attention over the past decade when outbreaks of the disease were found to be associated with severe neurological syndromes and congenital abnormalities. Unlike most other flaviviruses, ZIKV can spread through sexual and transplacental transmission, adding to the complexity of Zika pathogenesis and clinical outcomes. In addition, the spread of ZIKV in flavivirus-endemic regions, and the high degree of structural and sequence homology between Zika and its close cousin Dengue have raised questions on the interplay between ZIKV and the pre-existing immunity to other flaviviruses and the potential immunopathogenesis. The Zika epidemic peaked in 2016 and has affected over 80 countries worldwide. The re-emergence of large-scale outbreaks in the future is certainly a possibility. To date, there has been no approved antiviral or vaccine against the ZIKV. Therefore, continuing Zika research and developing an effective antiviral and vaccine is essential to prepare the world for a future Zika epidemic. For this purpose, an in-depth understanding of ZIKV interaction with many different pathways in the human host and how it exploits the host immune response is required. For successful infection, the virus has developed elaborate mechanisms to escape the host response, including blocking host interferon response and shutdown of certain host cell translation. This review provides a summary on the key host factors that facilitate ZIKV entry and replication and the mechanisms by which ZIKV antagonizes antiviral innate immune response and involvement of adaptive immune response leading to immunopathology. We also discuss how ZIKV modulates the host immune response during sexual transmission and pregnancy to induce infection, how the cross-reactive immunity from other flaviviruses impacts ZIKV infection, and provide an update on the current status of ZIKV vaccine development.

## Introduction

Zika virus (ZIKV) is a mosquito-borne arbovirus that was brought to attention in the past decade due to its link to serious neuropathogenesis in new-borns and adults. ZIKV is an enveloped, positive sense single-stranded RNA virus of the flavivirus genus in the Flaviviridae family, with a 50 nm diameter and a genome of about 10.8 kilobases in length (1). It was incidentally discovered in 1947 in the Zika Forest of Uganda, during a study on the vectors of sylvatic yellow fever. Until recent years, ZIKV never garnered much attention as there were only sporadic infections occurring in Africa and Asia (2), and the disease was thought to be either asymptomatic or mild and self-limiting. Symptoms and signs of ZIKV infection typically include low-grade fever, arthalgia, pruritic rash, conjunctivitis, myalgia, retro-orbital pain, headache, dysesthesia, and asthenia. Abdominal pain, diarrhea, nausea and mucous membrane ulcerations are some of the less common symptoms and signs (3–5). Thrombocytopenia has been reported, which could be due to an immune-mediated mechanism (6).

A string of outbreaks occurred since 2007 which eventually led to the observation of an increased incidence of new-borns with microcephaly and adults with Guillain-Barre syndrome in ZIKV-endemic populations. This prompted the World Health Organization (WHO) to declare ZIKV as a Public Health Emergency of International Concern (PHEIC) on 1 February 2016 to investigate the associations (7). These unique characteristics were not seen in infections by other similar viruses and has thus prompted researchers to investigate the mechanisms of ZIKV infection and develop preventative and treatment strategies against ZIKV infection.

Extensive research on ZIKV found that the effects on a new-born is not just limited to microcephaly, but also include various other adverse pregnancy outcomes such as miscarriage, fetal growth restriction, a range of fetal brain anomalies (ventriculomegaly, intracranial calcification), ocular abnormalities and hearing loss (8–11). In the developing mammalian brain, ZIKV efficiently targets the neural progenitor cells (NPCs), astrocytes, microglia and oligodendrocyte precursor cells (12). Studies on tissue tropism identified ZIKV from various tissues such as the placenta, fetal brain, male and female reproductive tract and body fluids, consistent with cases reporting vertical and sexual transmission. ZIKV RNA can persist for weeks in body fluids, and up to 6 months post-infection in seminal fluid (13). In adults, other than Guillain-Barré syndrome (GBS), very few cases of CNS involvement have been reported. Some of the complications include encephalitis, meningitis, myelitis, meningoencephalitis, transverse myelitis and neuropsychiatric symptoms (14–18). To characterize the cellular responses and molecular mechanisms involved in Zika pathogenesis, extensive virus-host interactome analyses have been conducted. These studies identified key cellular proteins that are associated with neuronal development and neurological diseases (19–21).

ZIKV infection is diagnosed by molecular and serological testing. For symptomatic individuals (≤7 days after onset of symptoms), ZIKV infections is established by real-time reverse transcription polymerase chain reaction (rRT-PCR) for ZIKV RNA in serum, urine or whole blood. If the rt-PCR results are negative, serologic testing: IgM ELISA and plaque reduction neutralization test (PRNT) are prompted. As for symptomatic individuals (>7 days after onset of symptoms), the diagnostics testing for ZIKV includes IgM and PRNT (22). However, cross-reactivity with other flavivirus antibodies complicates interpretation of serologic results. Although PRNT is generally the most specific, it may not be sufficient to distinguish between ZIKV and DENV infection (23). ZIKV NS1-sepcific IgM antibody approach was found to be more specific and was not detected in patients with previous Dengue (24). 

As of today, there are no approved vaccines or antivirals for ZIKV infection (25). With the low number of active cases, clinical trials on potential vaccines and antivirals were halted until they can be tested on more patients in a future outbreak (26). This review article aims to summarize the current knowledge on immune evasion strategies used by ZIKV leading to its pathogenesis to aid in the development of vaccine and therapeutics. There are various host defence mechanisms involved in the detection and elimination of ZIKV. These defences can be broadly categorized three categories, namely intrinsic, innate and adaptive immunity.

## ZIKV Replication Cycle and Targeted Host Factors

ZIKV like other viruses’ hijacks host cell machineries for its replication. Moreover, being an RNA virus with a small genome size, successful replication of ZIKV requires utilization of many host factors. In addition, small RNA viruses are highly adaptive, with a high degree of genetic variation in every cycle of replication. This presents a challenge for the development of an effective anti-ZIKV antiviral that target epitopes of ZIKV. Instead, perhaps host factors can be a better target for ZIKV therapeutics and vaccine, as they mutate at a much slower rate.

Entry of ZIKV into host cells is facilitated by the binding of ZIKV surface glycoproteins with host cell surface receptors. Expression of some host receptors have been found to increase ZIKV infectivity. These include TIM, TAM (Tyro3, Axl, Mertk) and DC-SIGN receptors (27). TIM and TAM are families of transmembrane receptors that recognizes and binds to phosphatidylserine (PS), which is a molecule which signals for phagocytosis of cells expressing it. PS signalling is a characteristic feature of apoptotic cells, but viruses such as ZIKV and DENV also express PS on its envelope as a disguise to gain access to host cells. TIM directly binds to PS, whereas TAM receptors require a bridging ligand Gas6 to bind PS (28). DC-SIGN is a receptor present on innate immune cells such as dendritic cells and macrophages. It binds to glycans and facilitates uptake of antigens expressing these glycans (29). Binding of ZIKV envelope glycoprotein to these receptors initiate clathrin-mediated endocytosis, which brings in the virus in a clathrin-coated endosome. Other cofactors on host cell membranes such as heat shock protein 70 (Hsp70) may also play a role in mediating ZIKV entry (30).

As the endosome matures, hydrogen ions are pumped in resulting in an acidic internal environment. The low pH triggers a structural change in the envelope of ZIKV, causing it to fuse with the endosomal membrane, releasing the viral genome into the cytoplasm (31). The ZIKV genome comprises a 5’ and a 3’ non-coding region, with a single open reading frame which encodes for three structural proteins – envelope (E) proteins, capsid (C) proteins, premembrane/membrane (prM/M) proteins and seven non-structural proteins – NS1, NS2A, NS2B, NS3, NS4A, NS4B, NS5. The structural proteins form structural components of virions and assemble new virus particles, and mediate virus entry and encapsidation. The E protein contains a transmembrane domain, and three ectodomains (EDI, EDII and EDIII) located outside the membrane. The protein is involved viral attachment, membrane fusion and cellular receptor binding. It also represents the main target for neutralising antibodies (1, 32). The non-structural proteins have a variety of functions which includes evasion of host immune response, alteration of host cell signalling pathways, and replication of ZIKV RNA, all of which contributes to effective viral replication and pathogenesis (1). The NS1 protein is essential for viral RNA replication, and the immune evasion and pathogenesis by interacting with host immune factors such as RIG-I-like receptors (33–35). It is involved in virus maturation through interaction with viral prM and E protein (20). NS1 also elicits production of protective antibodies (36). The NS3 and NS5 proteins, possess all enzymatic functions required for RNA replication. The NS3 protein consists of an N-terminal containing a serine protease domain (NS3pro), which is essential for proteolytic processing of the viral polyprotein, and a C-terminal bearing RNA helicase, RNA triphosphatase and RNA-stimulated NTPase domain that are critical for RNA replication (37, 38). ZIKV NS5 consists of an RNA dependent RNA polymerase (RdRp) domain, which is responsible for viral RNA synthesis, and a methyltransferase (MTase) domain, that is involved in translation and evasion of host immune response (34). 

The released positive-sense single-stranded RNA virus (+ssRNA) is then translated by the host cell’s translational machinery. Translation begins in the cytosol and is directed to the endoplasmic reticulum (ER) *via* ER-localizing signals on the nascent polypeptide chain. The polypeptide chain embeds and translocates into the ER with the help of Sec61 translocon, ER membrane complexes (EMCs), signal peptidases and oligotransferases (39). The completed polyprotein is subsequently cleaved by host signal peptidase and viral NS2B-NS3 protease complex into individual viral proteins, which then localizes to different components of the cell to carry out their respective functions (1).

At the ER, ZIKV enhances genome replication, virion assembly and transportation by remodelling the ER architecture, forming an assortment of virus-induced membrane structures, which includes vesicle packets, convoluted membranes, zippered ER and pancrystalline arrays (39). NS4A interacts with reticulon 3.1A, a host factor responsible for regulation of membrane structures, to induce curvature of the ER membrane, forming vesicles where ZIKV genome replication occurs. Knockdown of this host factor have been shown to reduce virus-induced structures and ZIKV replication (40).

For the maturation and eventual release of ZIKV virion, utilization of the host cell secretory machinery is required. Newly assembled virions go through a series of maturation processes in the golgi network. The acidic environment of the trans-golgi network once again induces a conformational change in the ZIKV E proteins from a spiky trimeric heterodimer to a flat homodimer. This exposes the furin cleavage site, enabling the cleavage of prM proteins into mature M proteins by furin (1), which is a host protease abundant in golgi bodies. Vesicles containing mature ZIKV then fuses with the plasma membrane to release the mature virions into the extracellular space.

## Host Intrinsic Defenses Against ZIKV

Intrinsic immunity are host defences that are constantly present in host cells. These defences detect and restrict viral replication *via* host cellular mechanisms such as autophagy, apoptosis, RNA interference/decay and formation of stress granules (41). Several studies have identified intrinsic defences that restrict ZIKV replication. Stress granules (SG) are collections of ribonucleoproteins made up of mRNA complexes stalled in the initiation phase of translation. This can be due to the phosphorylation of eukaryotic initiation factor eIF2α by kinases such as protein kinase R (PKR), PKR-like endoplasmic reticulum kinase (PERK) and general control nonderepressible (GCN) that are activated at times of cellular stress (42). Stress granule proteins G3BP, TIA-1 and TIAR are often targeted by viruses to inhibit SG formation. Flaviviruses such as DENV and WNV have been known to sequester TIAR and TIA-1 to be used for their RNA replication (43). Studies by Hou et al. and Amorim et al. highlighted ZIKV’s ability to inhibit phosphorylation of eIF2α, thereby preventing formation of stress granules and ensuring the continuity of viral replication (44, 45). However, Hou et al. also demonstrated inhibition of SGs formed *via* eIF2α-independent mechanisms by ZIKV in HFA and A549 cells (45), while Amorim et al. demonstrated ZIKV’s inability to inhibit SGs formed *via* eIF2α-independent mechanisms in Vero cells (44). Although ZIKV has the ability to prevent SG formation, both studies found that ZIKV infection did not significantly induce formation of SGs.

Reticulophagy on the other hand is another intrinsic defence mechanism that is likely to be more important in restricting ZIKV replication. As ZIKV modulates the ER to aid in its replication and assembly, ER degradation by reticulophagy can be an important step for host cells to inhibit ZIKV replication. Lennemann and Coyne demonstrated that inhibition of the reticulophagy receptor FAM134B resulted in a significant increase in ZIKV and DENV RNA levels. They also identified the ability of flavivirus NS2B3 to cleave FAM134B, disrupting the reticulophagy process (46).

## Host Innate Immune Response Against ZIKV

The innate immune system is activated by detection and subsequent phagocytosis of foreign antigens by innate immune cells such as dendritic cells and macrophages. Innate immunity plays an important role in restricting ZIKV pathogenesis, mainly through production of interferons (IFN) and interferon-stimulated genes (ISGs) which encodes various proteins that antagonize processes of ZIKV replication (47). For ISGs to be produced, a long, multistep immune signalling pathway is required. This process generally begins when ZIKV enters a host innate immune cell. Here, different types of pattern recognition receptors (PRRs) detect specific pathogen associated molecular patterns (PAMP). The RIG-I-like receptors (RLR) are PRRs present in the cytoplasm of these immune cells. RIG-I and MDA5 are RLRs that detect viral RNA molecules. Upon detection of RNA, the RLRs translocate to the mitochondria to activate the mitochondrial antiviral signalling protein (MAVS), which then sends a downstream signal to activate TANK-binding kinase 1 (TBK1), an enzyme required for the phosphorylation of transcription factors IRF3, IRF5, IRF7 and NF-κB. These activated transcription factors then translocate into the nucleus to aid in the production of interferons (48). Apart from the RLRs, toll-like receptors (TLR) are another type of PRR. The endosomal TLR3 and TLR7/8 function to detect viral dsRNA and ssRNA respectively. Once activated, TLR3 signals TRIF, which then activates TRAF6 and transcription factors IRF3, IRF 7 and NFkB. On the other hand, TLR7/8 signals MyD88, an adapter protein to activate transcription factors such as NF-κB and IRF7 (49). The third type of PRRs are NOD-like receptors (NLRs). Detection of PAMPs triggers oligomerization of NLRs and subsequent inflammasome formation. NLRP3 inflammasomes activate caspase 1, resulting in the activation of proinflammatory cytokines such as IL-1β and IL-18 (50). Another type of PRR involves the cGAS-STING pathway. This is a cytosolic DNA-sensing mechanism that detects damaged DNA and signals for downstream processes to mount a type I IFN response (51).

Type I interferons (IFN α and β) play an important role in the immune response towards flaviviruses. After the production of type I IFN *via* the multistep process initiated by detection of viral RNA by PRRs, the IFNs bind to IFN receptors consisting of two dimerized subunits - IFNAR1 and IFNAR2. Binding by type 1 IFN induces activation of Janus kinase (JAK), which phosphorylates signal transducers and activators of transcription 1 (STAT1) and 2 (STAT2). Phosphorylated STAT1 and STAT2 forms a heterodimer which associates with Interferon Regulatory Factor 9 (IRF9), forming the IFN-stimulated gene factor 3 (ISGF-3), which results in the increased expression of ISGs (47).

## Evasion of the Innate Immune Response by ZIKV

**Figure 1:**
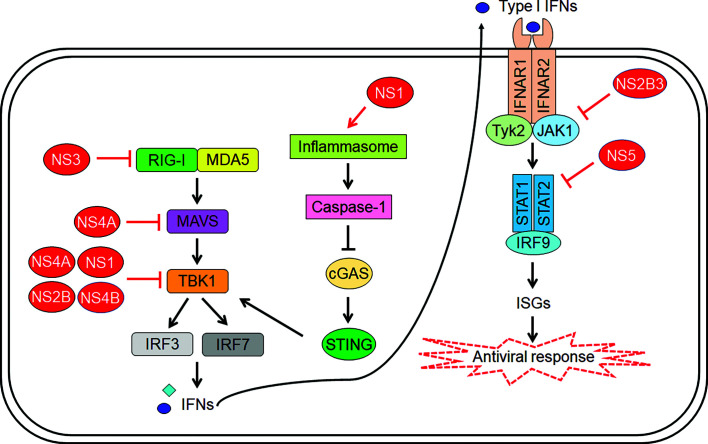
Zika virus (ZIKV)-mediated inhibition of host innate immunity. The viral proteins indicated in red color interfere with signalling pathways at multiple steps leading to suppression of type I interferon (IFN) induction as well as IFN-mediated expression of IFN-stimulated genes (ISGs).

To ensure successful replication, ZIKV employs several strategies to evade the host innate immune system. These evasion processes are carried out by ZIKV non-structural proteins, and they target various points of the IFN signalling pathway. At the RIG-I-like receptors, ZIKV NS3 binds to the 14-3-3 protein, preventing translocation of RIG-I and MDA5 to the mitochondria for the activation of MAVS (52). In addition, ZIKV NS4A was found to interact with MAVS, preventing its activation by RIG-I and MDA5 (53) (**Figure 1**). Co-immunoprecipitation studies by Wu et al. identified interactions between TBK1 and ZIKV NS1 and NS4B, which prevents TBK1 from phosphorylating transcription factors of IFN genes (54) (**Figure 1**). Another group reported suppression of TBK1 by NS1, NS2A, NS2B and NS4B (55) (**Figure 1**). As we move downstream, ZIKV NS5 has been shown to interact with IRF3, reducing the induction of IFN-β genes (55). Another mechanism is the ability of ZIKV NS1 to activate inflammasomes by preventing proteosomal degradation of caspase-1 (**Figure 1**). This seems contradictory to the function of inflammasomes but caspase-1 levels were found to be negatively correlated with type I IFN response, making high caspase-1 levels beneficial for ZIKV replication. The stabilization of caspase-1 as a result of inflammasome activation by NS1 was also found to facilitate cleavage of cGAS by caspase-1. This disrupts the cGAS-STING pathway, reducing the induction of type I IFN signalling downstream (56) (**Figure 1**).

At the JAK-STAT signalling pathway, ZIKV NS5 can bind to STAT2, facilitating its degradation by proteasomes (**Figure 1**). This is a species-specific process that occurs in human and nonhuman primate models but not in murine models (57), which is one of the reasons immunocompetent mice are resistant to ZIKV infection. Besides STAT2 degradation, NS5 was also found to inhibit phosphorylation of STAT1 and STAT2. Another mechanism is the ability of ZIKV NS2B3 to interact with and degrade JAK1, inhibiting phosphorylation of STAT and subsequent downstream processes (54) (**Figure 1**). Although ZIKV downregulates type I and type III IFN, it was found to upregulate type II IFN. Chaudhary et al. found that suppression of type II IFN signalling pathway led to decreased viral replication, and increased replication was observed in cells that were treated with IFN-γ before subjected to ZIKV (58). Selective degradation of STAT2 and sparing of STAT1 leads to the formation of STAT1 homodimers, which results in the activation of IFN-γ-stimulated genes, such as CXCL10. CXCL10 has shown to play an essential role in CD8 T cell recruitment during West Nile virus (WNV) infection in the central nervous system (59). Importantly it also has been associated to neuronal damage by causing apoptosis in fetal neurons (60). Therefore, the activation of type II IFN seems to play an active role in aiding ZIKV replication and may have implication on Zika neuropathogenesis.

## Role of the Adaptive Immune Response in ZIKV Infection

Several studies have identified the role of host adaptive immunity in response to ZIKV infection. ZIKV infection has shown to induce T cell activation in both humans and mouse models. Winkler and colleagues identified proliferation of CD4+ and CD8+ T cells peaking at 7 days post-infection in adult wild-type mice despite undetectable ZIKV levels. In the same study, depletion of CD4+ and CD8+ T cells only resulted in transient weight loss in immunocompetent mice but causes significant disease in mice with anti-IFNAR antibodies. Also, mice treated with anti-IFNAR antibodies without T cell depletion did not suffer from disease or experience weight loss despite increased viral replication, suggesting that these T cells play an important role in the restriction of ZIKV infection only when type I IFN response is compromised (61).

**Figure 2:**
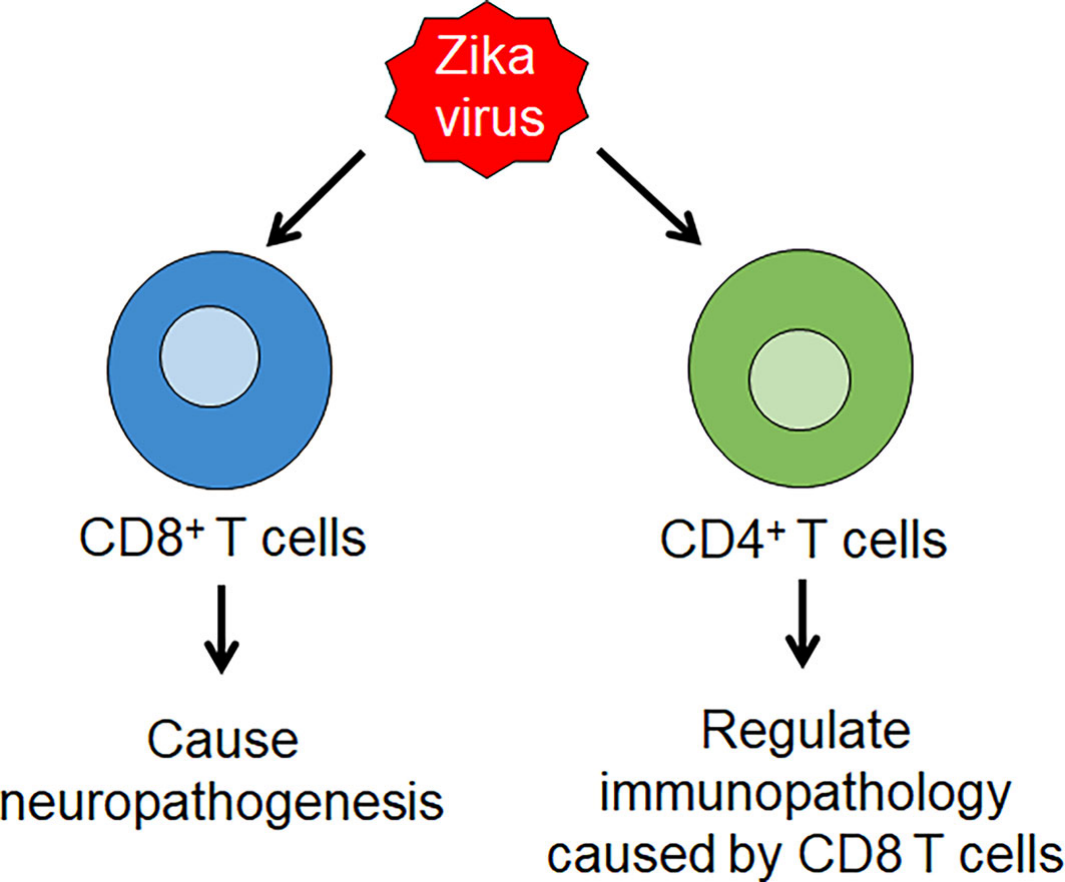
Zika virus (ZIKV) infection induces T cell immunity. ZIKV infection leads to the production of Th 1 CD4 T cell and effector CD8 T cell responses. CD8 T cells lead to ZIKV neuropathology, while CD4 T cells play a regulatory role through reduction of immunopathology caused by CD8 T cells.

CD4+ T cells were found to differentiate into Th1 cells during ZIKV infection as evidenced by the increased production of IFN-γ, IL-2 and TNF-α cytokines and transcription factor T-bet. Effector CD8+ T-cells are also found to produce IFN-γ and TNF-α, and CD8+ levels are increased in ZIKV infection, accompanied with increased expression of granzyme B on them (62). A study using IFNAR KO mice demonstrated the role of T Cells in ZIKV Pathogenesis. Depletion of CD8+ T cells resulted in an increase in viral load in the brain but exhibited improved survival and reduced paralysis. On the other hand, depletion of CD4+ T cells alone caused paralysis in all mice, while depletion of both CD4+ and CD8+ T cells resulted in an intermediate phenotype with an increase in survival and reduction in paralysis (63). Another study showed that depletion of CD4+ T cells caused high viral load in the CNS and decreased survival (64). These findings suggest the role of CD8 T cells in causing neuropathology and potential regulatory role of CD4 T cells through reduction of immunopathology caused by CD8 T cells (**Figure 2**). In addition, it was also found that depletion of CD8+ T cells led to an increase in ZIKV-positive neurons and it was shown that CD8 T cells mediate lysis of ZIKV-infected neurons. These observations suggest that CD8 T cells limit ZIKV replication within the neurons, but cause neuropathogenesis leading to paralysis (**Figure 2**).

In terms of humoral immunity, B cell response to ZIKV have been elicited by the detection of neutralizing antibodies in mice models (62). CD4 T cells have shown to be essential for production of ZIKV-specific humoral response by driving antibody production (65). In humans, Lai et al. detected anti-ZIKV IgM, ZIKV-specific plasmablasts and neutralizing antibodies in ZIKV-infected patients’ sera (66). The majority of the neutralizing mAbs derived from memory B cells of ZIKV-infected patients were found to primarily target EDIII or quaternary epitopes of whole ZIKV. In different mouse models, the neutralizing EDIII-specific antibodies have shown to provide protection against lethal infection of ZIKV (67, 68). Researchers have also explored the effects of cross-reactivity between ZIKV and DENV. Monoclonal antibodies isolated from plasmablasts of patients with past DENV infection were also found to cross-react with ZIKV, binding to components of ZIKV but are mostly ineffective in neutralizing it. Potent neutralizing ability was mostly limited to type-specific antibodies (69). This brings about the possibility of antibody-dependent enhancement, which was elicited in a few studies (67, 70), where poorly neutralizing antibodies bind to and bring viruses to infect immune cells expressing Fc receptors, increasing virion production and worsening disease outcomes.

## Zika Autoimmunity and Guillain-Barré Syndrome

A recent systematic review and meta-analysis characterized ZIKV-associated GBS as a broad sensory demyelinating illness with frequent facial paralysis and a severe disease course (71). A study found that ZIKV patients with GBS had significantly higher levels of anti-ganglioside autoantibodies compared to Zika patients without GBS (72). As anti-gangliosides have been implicated in the pathogenesis of GBS (73), these autoantibodies could be a factor in ZIKV-linked GBS. In several ZIKV-infected patients with GBS, mononuclear lymphocytic infiltration and demyelination associated with inflammation have been observed (74).

## Role of the Immune Response in Sexual Transmission of ZIKV

Zika virus (ZIKV) is transmitted to humans primarily by *Aedes* mosquitoes. However, several evidence supports that ZIKV can be transmitted sexually (75, 76). There is no evidence of sexual transmissibility in any other flaviviruses. Sexual transmission poses a serious threat to humans who are outside of the ZIKV epidemic-prone locations. Several studies found high titers of ZIKV RNA in human seminal fluid for months after clearance of viremia (77, 78). In addition, the presence of ZIKV in seminal fluid is more robust than in vaginal fluids (79). In males, the immunological responses facilitate the persistent infection of the MRT, while in females, a robust immune response controls the virus infection (80, 81). In line with this, male-to-female transmission is more common than female-to-male transmission (82, 83)

ZIKV has shown strong tropism for the male reproductive tract MRT. In animal models, ZIKV-infected Sertoli cells, spermatogonia, primary spermatocytes, Leydig cells, peritubular myoid cells, and epithelial cells of the lumen (84, 85). While in the human testis *ex vivo* model, ZIKV was found to infect somatic and germ cells, including Sertoli cells, testicular macrophages, Leydig cells and peritubular cells (86). The inflammatory response that occurs following ZIKV infection mediate the recruitment of innate cells (myeloid DCs, non-classic monocytes, and NK cells) as early targets of the virus and subsequently, the virus travel in these cells to the testis. To reach the testis, it is likely that ZIKV manipulates the antiviral mechanism to disrupt the blood testicular barrier (BTB) junctions, or the virus uses its proteins to destroy the junctions to cross the barrier (80, 87, 88).

The Sertoli cells, which consist of the blood-testis barrier (BTB) have shown to be highly susceptible to ZIKV infection. ZIKV has been shown to effectively infect Sertoli cells for the long-term without causing cell death (86, 89, 90). Infection of Sertoli cells induces pro-inflammatory mediators and genes linked to multiple innate immune response pathways. Among these, the IFN signaling was most significantly modulated. Down-regulation of adherent junction genes were also observed (90). These findings suggest that ZIKV can induce a strong antiviral response in Sertoli cells, which can alter the permeability of BTB.

Another study demonstrated that ZIKV infection of Sertoli cells induced cell adhesion molecules (VCAM-1 and ICAM-1), which mediate the adhesion of naive immune cells to Sertoli cells and increase BTB permeability. In addition, ZIKV infection in macrophages results in the production of inflammatory cytokines and chemokines that can degrade the tight junction protein ZO-1 and directly affect the integrity of the BTB (87). Proteomic analyses of ZIKV-infected human Sertoli cells revealed dysregulation of different proteins and cellular signaling pathways involved in the maintenance of Sertoli cells tight junction permeability and BTB, spermatogenesis as well as testicular development. Mitogen-activated protein kinase (MAPK)/extracellular signal-regulated kinase (ERK), Metalloproteinase inhibitor 1 (TIMP1), IL-6, Stanniocalcin-1 (STC1), nuclear factor-κB (NF-κB), insulin-like growth factor 1 (IGF1) and fibroblast growth factor (FGF) were among them (91)

ZIKV infection elicited virus-specific IgG in the lumen of the vagina and recruited virus-specific T cells (CD4+ and CD8+ T cells) to the female reproductive tract (FRT). Transfer of virus-specific IgG and circulating memory T cells reduced viral replication, with the humoral response providing greater protection compared to cellular response. These findings suggest that the humoral and cellular responses confer protection against intravaginal ZIKV infection (81). Overall, these findings support the dual role of the immune response in the pathogenesis of ZIKV sexual transmission: a promoter of viral dissemination in males and as a protective factor in females.

## Relationship Between Immunological Response During Pregnancy and ZIKV Infection

It is generally known that the immune system changes during pregnancy, whereby it must be finely balanced to avoid fetal and placental rejection and protect the fetus from infections. During pregnancy, hormones play a critical role in modulating the immune response by lowering the antigen-presenting potential of dendritic cells (DCs), reducing the number of monocytes and macrophages, as well as blocking natural killer cells, T and B cells (92). Evidence supports the production of both pro-inflammatory and anti-inflammatory factors at different stages of pregnancy (93). ZIKV can cross the placental barrier and has been shown to cause damage in the decidua and chorionic villi of the placenta. The virus has been shown to infect the placental cells such as cytotrophoblast (CTB), endothelial cells (En), syncytiotrophoblast (STB), mesenchymal cells (MS), fibroblasts (Fi), Hofbauer (Hf), macrophages and decidual cells (94–96). ZIKV can persist in the placenta for a long time after the onset of maternal symptoms and may serve as a latent viral source of fetal infection (94, 95, 97).

In a mouse model, infection with ZIKV at a gestational age (similar to the mid and late first trimester in humans) showed that type I IFN led to detrimental effects in pregnancy. IFNAR signaling in the conceptus reduced ZIKV replication in the placenta. However, IFNAR signaling mediated apoptosis of fetal endothelial cells and trophoblasts, suppressed the development of the placental labyrinth, disrupted the maternal-fetal blood barrier, fetal hypoxia and subsequently contributing to severe intrauterine growth restriction and fetal demise (98). Activation of type I IFN induced by ZIKV infection leads to significant neuroinflammation and tissue injury.

ZIKV infection during pregnancy has been shown to induce production of pro-inflammatory cytokines (IFN-γ, IFN-α, IL-6 and IL-17A) and chemokines (CXCL10, CCL2, CXCL9, and CXCL8). Expression of cytokines such as TNF-α and IFN-γ were observed in placental tissues (96). Increased levels of CXCL10, IL-22, MCP-1, and TNF-α were seen in pregnant women carrying babies with brain anomalies (99). Oher than that, higher levels of CCL2, CXCL10, IL-6, IL-8 and VEGF in amniotic fluid of ZIKV-infected pregnant women who gave birth to microcephaly babies (100). The increase in CCL2, CCL3, CCL4, CXCL9, and CXCL8 has been linked to neurological damage and fetal abnormalities (100, 101). The elevated level of CCL2 and its inverse correlation with the levels of CD163, TNFRSF1A, and CCL22 were linked to ZIKV-induced abnormal birth. In addition, distinct subsets of cytokines were detected at different trimesters. Notably, most of these cytokines are involved in the infiltration of leukocytes, in particular NK cells and monocytes, which are associated with pregnancy outcomes (101).

ZIKV-infected pregnant mice displayed increased levels of DCs associated with immunotolerance, reduced levels of cells expressing pro-inflammatory IL-12 and lower levels of Ag-experienced CD8+ T cells. These findings suggest that the immunotolerance during pregnancy may hinder efficient activation of the antiviral T cell response (102). In another study, ZIKV-infected pregnant mice caused a significant reduction of proliferating CD4+ and CD8+ T cells compared to ZIKV-infected non-pregnant mice. Furthermore, ZIKV-infected pregnant mice had considerably less granzyme B positive CD8+ T cells, indicating a loss in cytolytic effector activity (61). Hence, it is evident that pregnancy alters of the innate and adaptive immune responses to ZIKV infection. However, no significant differences were observed in the level of neutralizing antibodies between pregnant and non-pregnant mice, suggesting that pregnancy may not significantly alter the humoral immune response to ZIKV (61).

## Cross-Reactive Immunity Between ZIKV and Other Flaviviruses

The structure of ZIKV E protein, which contains EDI, EDII, and EDIII, is similar to the E protein of other flaviviruses, including DENV, WNV and YFV. The amino acid sequence E proteins of ZIKV and DENV type 2 share an antigenic similarity of 53.9% (103). The high sequence similarity in the E protein between ZIKV and other flaviviruses, particularly DENV, raises a major concern of cross-reactivity as well as antibody-dependent enhancement (ADE). Various studies have demonstrated that monoclonal antibodies (mAb) against DENV and sera from DENV patients can both enhance and neutralize ZIKV infection *in vitro* and *in vivo* (104–107).

ZIKV infection in individuals previously exposed to DENV induced a rapid and strong plasmablast response, mostly originating from the memory B cell compartment. The acute B cell response to ZIKV exhibited preferential binding and neutralization of DENV, providing evidence of original antigenic sin (OAS). Although most of the antibodies were broadly cross-reactive, they were poorly neutralizing against ZIKV and enhanced its infection, supporting that pre-existing immunity to DENV may have a deleterious impact on early protective B cell responses to ZIKV (69). In pregnant mice, pre-existing DENV antibodies increased vertical transmission, enhanced ZIKV replication in placenta and resulted in adverse outcomes. The enhancement effect observed in these pregnant mice was dependent on Fc receptors (108, 109). In contrast, ZIKV infection of nonhuman primates immunized with DENV or YFV did not cause an increase in ZIKV titer or adverse clinical outcomes despite modulation of the immune response (110). A cohort of individuals from Salvador (epicenter of ZIKV epidemic in Brazil) showed that pre-existing antibodies against DENV contributed to decreased risk of ZIKV infection and symptoms (111). In another cohort study involving pregnant women infected with ZIKV, previous DENV infection did not cause disease severity and abnormal birth outcomes (112).

In a mouse model, administration of low concentrations of DENV-immune plasma, displayed enhancement of ZIKV infection and resulted in a higher mortality rate. However, at higher concentrations, mice displayed milder symptoms and 100% survival after ZIKV infection (70). Therefore, the cross-reactivity or cross-protection against ZIKV is likely dependent on the concentrations of the anti-DENV antibodies. Another important factor determining the antibodies’ protective effects on DENV is the time interval between ZIKV infection and previous dengue fever incidence. A study found that the protective effect could last up to 6 years (113). A study proposed that the cross-protection against congenital Zika syndrome (CZS) conferred by DENV neutralizing antibodies was not dependent on the antibody titer but likely mediated by the immune response (114). As observed in humans, DENV antibodies have been shown to increase CD4+ and CD8+ T-cell responses during ZIKV infections (115, 116). Also, in pregnant mice, DENV cross-reactive CD8+ T cells have been demonstrated to protect the fetus during ZIKV infection (117, 118).

The cross-reactivity effects induced by pre-existing antibodies against other flaviviruses, may represent a challenge for the development of vaccine for ZIKV. Pre-existing antibodies may produce a strong immune response against the ZIKV vaccine through cross-reactive T cells, thus enhancing the vaccine efficacy (65). However, vaccination against ZIKV could also lead to ADE of DENV infection and vice versa, enhancing virus entry and potentially resulting in severe disease. Increasing evidence suggests that previous ZIKV immunity leads to increased DENV infection and risk for severe dengue (119, 120). Hence, designing an ideal vaccine for ZIKV requires careful consideration of potential ADE.

## Zika Virus Vaccine Development

Nearly half of the world’s population lives in regions at risk of Zika transmission, and the possibility of future Zika epidemics remains high (121). A mathematical model based in Nicaragua has predicted a possible ZIKV outbreak in 2047, affecting mainly the women of reproductive age. However, if protective immunity wanes, ZIKV recurrence may happen sooner (122). With the association between ZIKV and adverse pregnancy outcomes, congenital diseases, and severe developmental problems, the search for an effective vaccine has become even more urgent.

The WHO has proposed two strategies for ZIKV vaccine development roadmap: emergency outbreak response and endemic use. The emergency outbreak response entails mass vaccination of pregnant women and women of child-bearing age to prevent ZIKV-related adverse pregnancy outcomes and neurological complications. On the other hand, endemic use involves a broad vaccination of the general population and routine immunization to establish population immunity and prevent ZIKV transmission (123). Several ZIKV vaccine candidates under various platforms have been developed over the past 5 years. These include inactivated, live attenuated, viral-vectored, subunit, nucleic acid and messenger RNA (mRNA) vaccines. Some of these vaccines have demonstrated promising results in preclinical studies and have advanced into clinical trials. In animal models, a number of vaccine candidates were found to reduce ZIKV RNA levels in maternal, placental, testis and fetal tissues, and subsequently prevent their damage (124–126). Another study demonstrated the ability of live-attenuated ZIKV vaccine candidates to prevent viral transmission during pregnancy and protect against testis infection and injury in mice (125). As of September 2021, 12 ZIKV vaccine candidates are in clinical evaluation (**Table 1**) (ClinicalTrials.gov).

**Table 1:** Zika vaccine candidates in clinical trials.

Vaccine name	Immunogen	Sponsor name	Clinical trial (Status)	Clinical trial results	Ref.
DNA vaccines					
GLS-5700	prM/E	GeneOne Life Science, Inc.	Phase 1 NCT02809443 (Completed)	No serious adverse events in healthy adults; all participants developed ZIKV-specific binding antibodies after third dose; cellular responses peaked at week 36 during follow-up	(127)
VRC5283	prM/E	NIAID	Phase 2 NCT03110770 (Completed)	Not reported	
	prM/E	NIAD	Phase 1 NCT02996461 (Completed)	Safe and well tolerated in healthy adults; produced detectable cellular responses and neutralising antibody responses against ZIKV proteins	(128)
VRC 5288	prM/E	NIAID	Phase I/Ib NCT02840487 (Completed)		
mRNA vaccines					
mRNA-1325	prM/E	ModernaTX, Inc.	Phase 1 NCT03014089 (Completed)	Not reported	
mRNA-1893	prM/E	ModernaTX, Inc.	Phase 2 NCT04917861 (Recruiting)	Not reported	
		ModernaTX, Inc.	Phase 1 NCT04064905 (Completed)	Not reported	
Live attenuated vaccines					
rZIKV/D4Δ30-713	prM/E	NIAID	Phase 1 NCT03611946 (Recruiting)	Not reported	
MV-ZIKA	prM/E	Themis Bioscience GmbH	Phase 1 NCT02996890 (Completed)	Not reported	
MV-ZIKA-RSP	prM/E	Themis Bioscience GmbH	Phase 1 NCT04033068 (Completed)	Not reported	
ChAdOx1	prM-E	University of Oxford	Phase 1 NCT04015648 (Recruiting)	Not reported	
		University of Oxford	Phase 1 NCT04440774 (Recruiting)	Not reported	
Purified Inactivated vaccines					
ZPIV	Whole Virus	Kathryn Stephenson	Phase 1 NCT02937233 (Completed)	Safe and well tolerated in healthy adults through 52 weeks; no serious or serious adverse event grade 3 related adverse events; vaccine immunogenicity required two doses	(129)
		NIAID	Phase 1 NCT02963909 (Completed)	Well tolerated and elicited robust neutralizing antibody titers in healthy adults	(129, 130)
		NIAID	Phase 1 NCT02952833 (Completed)		
		NIAID	Phase 1 NCT03008122 (Completed)	Not reported	
VLA1601	Whole Virus	Valneva Austria GmbH	Phase 1 NCT03425149 (Completed)	Not reported	
(TAK-426)/PIZV	Whole Virus	Takeda	Phase 1 NCT03343626 (Completed)	Well tolerated in adults with or without serological evidence of previous exposure to flaviviruses; no deaths, no vaccine related serious adverse event; mild to moderate adverse events reported; immunogenic in both flavivirus-naive and flavivirus-primed participants; seroconversion rates, PRNT responses were higher in flavivirus-naive than flavivirus-primed participants after both vaccinations.	(131)

ZIKV, Zika Virus; NIAID, National Institute of Allergy and Infectious Diseases; VRC, Vaccine Research Center; ZPIV, ZIKV purified inactivated virus vaccine; RVP, reporter virus particle; VRC, Vaccine Research Center.

NHPs with immune responses primed by infection with an African ZIKV strain were protected when re-challenged with an Asian ZIKV strain (132). Hence, a ZIKV vaccine based on a single strain could be adequate and protect against other strains. Since ZIKV causes severe birth outcomes and congenital malformations, the ability of the vaccines to adequately protect pregnant women, women of reproductive age and children need to be determined. To protect the fetus from ZIKV infection, protective immunity needs to be achieved during the first trimester or early second trimester. Importantly, the safety of the vaccines during pregnancy is required to be critically accessed. Another hurdle is the potential impact of the pre-existing flavivirus antibodies in people who have been previously infected or vaccinated against other flaviviruses. In light of this, a study conducted by Larocca et al. demonstrated that pre-existing DENV immunity did not reduce the immunogenicity or protective immunity of ZIKV candidate vaccines in animal models (rhesus macaques and mice) (133). However, the designing and administration of ZIKV vaccines require extreme caution, and their safety and immunogenicity need to be evaluated both in flavivirus-exposed and naïve populations.

## Conclusion

Zika virus (ZIKV) has been shown to modulate both the innate and adaptive immune system of the host defence mechanism to buy time to effectively induce infection and cause neuropathogenesis. Identification and understanding of the role of effector molecules in antiviral response and signal transduction pathways are crucial to decipher the host-ZIKV interactions. ZIKV uses multiple mechanisms to evade the host immune response. The majority of these mechanisms are triggered by the non-structural proteins. It modulates the innate immune response primarily by inhibiting the expression of type I IFNs and ISGs. Suppression of type I IFN increases dependency on adaptive immune response in circumventing infection.

Importantly, the ZIKV-induced host immune response is dependent on several factors: gender, pregnancy and pre-existing immunity against other flaviviruses. In males, the immune response facilitates ZIKV persistence through inflammatory factors and alteration of BTB permeability. While in females, the immune response seems to control ZIKV virus infection. However, the ZIKV cellular targets in the reproductive organs and the underlying mechanisms need to be further investigated. Alterations of the innate and adaptive immune responses during pregnancy have demonstrated to have significant effects on ZIKV infection and pathogenesis. Activation of type I IFN along with the expression of proinflammatory cytokines and impaired T cell response in ZIKV-infected pregnant women could be the major factors contributing to adverse fetal and birth outcomes. Given the important effects of pregnancy-induced immune modulation on ZIKV infection and the possible adverse outcomes, this area of research requires thorough understanding. This is also critical for successful vaccine development and administration in women of reproductive age and pregnant women. In addition, follow-ups of children born with *in utero* ZIKV exposure may aid in assessing the effects caused by the immune response and providing support for early intervention to improve the neurodevelopment of the children.

The effects of pre-existing flavivirus antibodies on ZIKV immunopathogenesis and their potential impact on Zika vaccine development have been a major concern. Various studies have demonstrated that pre-existing flavivirus immunity, particularly against DENV can both enhance and neutralize ZIKV infection *in vitro* and *in vivo*. The detrimental or protective effects of the pre-existing immunity is likely determined by the concentration of antibodies, time interval between exposure with different flaviviruses, and immune response. The majority of the studies conducted *in vitro* and in mice showed enhanced ZIKV replication and disease severity due to pre-existing flavivirus immunity. However, most of the investigations in nonhuman primates and humans showed the opposite and suggest that high rates of immunity may present a barrier to future ZIKV outbreaks. Other factors such as the ZIKV strains, the type of animal models used, and Fc receptors may also contribute to the effects of pre-exiting flavivirus antibodies. Future studies in nonhuman primates and humans are necessary to fully understand the effects of pre-existing antibodies on ZIKV infection, particularly in the placenta and also their impact on Zika vaccines. Studies also need to address the effects of Zika vaccines on DENV infection and potential ADE. Dissecting the dynamics of pre-existing flavivirus immunity will aid in understanding flavivirus pathogenesis and vaccine development.

## Author Contributions

Conceptualization by LL and VB. Methodology by LL and TK. Writing—original draft preparation by LL and TK. Writing, review and editing by LL, TK, NA, WJ, and VB. Supervision by VB and WJ. All authors contributed to the article and approved the submitted version.

## Conflict of Interest

The authors declare that the research was conducted in the absence of any commercial or financial relationships that could be construed as a potential conflict of interest.

## Publisher’s Note

All claims expressed in this article are solely those of the authors and do not necessarily represent those of their affiliated organizations, or those of the publisher, the editors and the reviewers. Any product that may be evaluated in this article, or claim that may be made by its manufacturer, is not guaranteed or endorsed by the publisher.
